# How autochthonous microorganisms influence physiological status of Zea mays L. cultivated on heavy metal contaminated soils?

**DOI:** 10.1007/s11356-018-3923-9

**Published:** 2018-12-18

**Authors:** Szymon Rusinowski, Alicja Szada-Borzyszkowska, Paulina Zieleźnik-Rusinowska, Eugeniusz Małkowski, Jacek Krzyżak, Gabriela Woźniak, Krzysztof Sitko, Michał Szopiński, Jon Paul McCalmont, Hazem M. Kalaji, Marta Pogrzeba

**Affiliations:** 1 Institute for Ecology of Industrial Areas, 6 Kossutha Street, 40-844 Katowice, Poland; 2 Department of Plant Physiology, Faculty of Biology and Environmental Protection, University of Silesia in Katowice, 28 Jagiellońska Street, 40-032 Katowice, Poland; 3 Department of Botany, Faculty of Biology and Environmental Protection, University of Silesia in Katowice, 28 Jagiellońska Street, 40-032 Katowice, Poland; 4 College of Life and Environmental Sciences, Exeter University, Exeter, UK; 5 Department of Plant Physiology, Warsaw University of Life Sciences SGGW, 159 Nowoursynowska Street, 02-776 Warsaw, Poland

**Keywords:** Indigenous microorganisms, Arbuscular mycorrhiza, Photosynthesis, Heavy metals, Oxidative stress, Mineral nutrients

## Abstract

The aim of this study was to investigate the effect of autochthonous microorganisms present in soil collected from heavy metal (HM) uncontaminated (Pb ≈ 59 mg kg^−1^, Cd ≈ 0.4 mg kg^−1^, Zn ≈ 191 mg kg^−1^), moderately (Pb ≈ 343 mg kg^−1^, Cd ≈ 12 mg kg^−1^, Zn ≈ 1876 mg kg^−1^), and highly (Pb ≈ 1586 mg kg^−1^, Cd ≈ 57 mg kg^−1^, Zn ≈ 3280 mg kg^−1^) contaminated sites on *Zea mays* elemental composition, physiological status, and growth parameters. For this purpose, half of the collected soil was sterilized and soil characterization was performed. After 45 days of cultivation, the presence of HM in the soil negatively affected photosynthesis and transpiration rates, relative chlorophyll content, anthocyanins index, chlorophyll fluorescence parameters, and content of oxidative stress products (H_2_O_2_ and Malondialdehyde) of *Zea mays*, while soil sterilization had a positive effect on those parameters. Average percentage of colonization of root segments by arbuscular mycorrhiza fungi decreased with an increase of HM contamination in the soil. The increase in shoot concentration of HMs, particularly Cd and Zn, was a result of contaminated soils sterilization. Aboveground biomass of maize cultivated on sterilized soil was 3-fold, 1.5-fold, and 1.5-fold higher for uncontaminated, moderately contaminated and highly contaminated soils respectively when compared to nonsterilized soils. Contrary to our expectation, autochthonous microflora did not improve plant growth and photosynthetic performance; in fact, they had a negative effect on those processes although they did reduce concentration of HMs in the shoots grown on contaminated soils.

**Electronic supplementary material:**

The online version of this article (10.1007/s11356-018-3923-9) contains supplementary material, which is available to authorized users.

## Introduction

The interaction between soil microbes and plant roots has been studied widely. The dynamics of this interaction depend on several factors, such as the physiological characteristics of plants and microorganisms, physicochemical soil properties, and even climatic conditions (Haney et al. 2015; Köhl et al. 2016; Mohite 2013; Pii et al. 2015). Plant associated microorganisms can play an important role in growth and nutritional status improvement (Cabral et al. 2016; Ercoli et al. 2017; Valliere and Allen 2016), as well as in the detoxification of harmful substances and maintenance of soil structure (Sułowicz et al. 2011; Thijs et al. 2014; Watts-Williams et al. 2017). Indigenous microorganisms living on heavy metal-contaminated sites have often adapted well to the presence of these elements, driven by long-term exposure to site-specific stress factors, and these adaptations can provide useful opportunities for bioremediation at such sites (Giller et al. 2009; Oliveira et al. 2014; Touceda-González et al. 2017; Vivas et al. 2003; Yu et al. 2014).

Many authors reported a connection between reduction of dehydrogenase activity and presence of pollutants in soil at high concentrations, which correlates with a decrease in microbial community diversity and biomass (Baćmaga et al. 2015; Wang et al. 2014; Wolińska and Stępniewska 2012).

An important component in the rhizosphere are the arbuscular mycorrhizal fungi (AMF), belonging to the phylum *Glomeromycota*, which live in symbiosis with 70–90% of plant species (Bonfante and Genre 2010; Bothe et al. 2010; Gianinazzi et al. 2010; Huang et al. 2009; Zhu et al. 2012). Recently, an increasing number of reports have emphasized the role of mycorrhiza in reducing stress reactions associated with environmental pollution by heavy metals (Firmin et al. 2015; Gucwa-Przepióra 2012; Li et al. 2016). AMF associated with plants may contribute to the accumulation of heavy metals in roots in a nontoxic form inside hyphal cell walls or bind them into phosphate compounds inside the fungal cells (Andrade and da Silveira 2008).

High concentrations of heavy metals in the soil negatively affect many plant physiological processes through the production of reactive oxygen species (ROS). This in turn results in lipid peroxidation, mostly irreversible oxidation of proteins and DNA damage. There are many reports indicating that plant-microbial interactions can alleviate oxidative stress caused by toxic concentrations of heavy metals (Islam et al. 2014; Islam et al. 2016; Nadeem et al. 2014). Islam et al. (2016) observed improving gas exchange parameters, such as photosynthetic rate (A), transpiration rate (E), stomatal conductance (g_s_), and intercellular CO_2_ concentration (C_i_), as well as reduction of H_2_O_2_ and malondialdehyde (MDA) content in lentils (*Lens culinaris* Medik) grown in heavy metal-contaminated soil inoculated with microorganisms, in comparison with non-inoculated ones.

To date, most experiments have investigated the influence of selected microbes on plant growth and development, considering either selected strains alone on sterilized soil or a combined effect of selected microbes with autochthonous microorganisms on nonsterilized soil (Bai et al. 2008; Guo et al. 2013; Liang et al. 2009; Rostami et al. 2016; Wang et al. 2007; Verma et al. 2014; Zhu et al. 2010). However, the influence of autochthonous microorganisms, particularly on plant growth and physiological processes, is poorly described in the literature (Liu et al. 2014; Miransari et al. 2009; Yang et al. 2015). Experiments conducted on sterilized and nonsterilized soil without inoculation can offer an opportunity to assess the impact of these autochthonous microorganisms on plants cultivated on soils with different levels of heavy metal contamination. We hypothesize that presence of autochthonous microorganisms in soils should improve plant growth and photosynthesis performance of *Zea mays* plants. Thus, the aim of this study was to investigate the relationships between plant growth and the chlorophyll *a* fluorescence parameters, gas exchange parameters, pigments content, mineral nutrient status, heavy metals concentration, and oxidative stress in maize plants cultivated on contaminated and noncontaminated soils with and without autochthonous microorganisms.

## Materials and methods

### Site description and soil sampling

Soil for the pot experiment was sampled from three experimental sites located in Upper Silesia, Poland. Sites were selected according to heavy metal (HM) contamination status: fallow land in Piekary Śląskie (50° 22′ 06.2” N 18° 57′ 52.9″ E) with a high degree of pollution (HM_high_), caused by close vicinity to the former Pb/Zn smelter; arable land in Bytom (50° 20′ 41.9” N 18° 57′ 19.9″ E) with a moderate level of contamination (HM_mod_), resulting from being a short distance (about 2.5 km) from the same smelter and arable land in Gliwice (50° 15′ 16.9” N 18° 40′ 46.6″ E), located about 24 km from the smelter, classified as a nonpolluted area (HM_low_). The zinc and lead smelter was operated from 1927 until 1990 when significant soil contamination occurred due to dust fall of particles containing HM (Fig. S1). At the Bytom site, cereals have been cultivated commercially for the last 20 years, while at the Gliwice site between 1970 and 2000, vegetables were cultivated without the use of any chemical fertilizer, and from 2000, this field was left fallow. The soil collected from each site was air-dried, passed through a 4-mm sieve, which removed stones and plant residues, mixed and divided into two parts. Half of each soil sample remained unsterilized while the second was sterilized in an autoclave at 120 °C and 1.2 bar for 20 min; this sterilization procedure was repeated four times with a 1-day interval.

### Plant growth experiment

Subsamples for each soil were put into plastic pots. For each variant, four pots (0.0015 m^3^ volume) were established. Experimental treatments were as follows:

- G_NS—nonsterilized soil from Gliwice, (HM_low_)
- G_S—sterilized soil from Gliwice, (HM_low_)
- B_NS—nonsterilized soil from Bytom, (HM_mod_)
- B_S—sterilized soil from Bytom, (HM_mod_)
- P_NS—nonsterilized soil from Piekary Śląskie, (HM_high_)
- P_S—sterilized soil from Piekary Śląskie, (HM_high_)

Caryopses of maize (*Zea mays* L., cv. ‘Lokata’) were germinated in the dark at 28 °C. After 2 weeks of germination, three seedlings per pot were planted. The plants were cultivated in a phytotron for 45 days under controlled conditions: temperature 24 °C, light intensity PAR = 300 μmol (photons) m^−2^ s^−1^ and humidity 40%. The measurements of plant gas exchange parameters and pigment content were conducted after the 18th day from planting and consequently repeated every 2 weeks until the end of the experiment. Additionally, at the end of the experiment, chlorophyll *a* fluorescence and plant height measurements were taken. Following this, plant and soil samples were collected for further analysis. The plants were divided into shoots and roots, washed in tap water to remove soil particles, and then washed again with deionized water. The rest of the plant growth measurements were then carried out along with assessments of malondialdehyde (MDA) and hydrogen peroxide (H_2_O_2_) contents in leaf samples. In addition, the intensity of root colonization by arbuscular mycorrhiza fungi was assessed. The rest of the shoots and roots biomass was dried for 72 h at 70 °C and then ground for elemental analysis. Soil samples collected from the pots after the experiment were examined for dehydrogenase activity.

### Soil physicochemical parameters

Soil samples were collected before the experiment, air-dried and sieved to 2 mm (for pH, electrical conductivity, organic matter content) and then ground to < 0.25 mm for further analysis (total metal concentration, bioavailable metal concentration, nutrients: N, P, K).

Soil pH was measured in H_2_O (ratio 1:2.5 *m*/*v*) with a combination glass/calomel electrode (OSH 10-10, METRON, Poland) and a pH-meter (CPC-551, Elmetron, Poland) at 20 °C. The electrical conductivity was determined using the same device as for pH measurements using an ESP 2ZM electrode (EUROSENSOR, Poland) following the standard Polish protocol, PN-ISO 11265:^1997^.

Soil texture was evaluated by the sieve method according to protocol ISO 11277:^2009^. Soil organic matter content (OM) was measured by loss on ignition as follows: air-dry soil was dried at 105 °C for 24 h and then (5 g) treated with 550 °C for 4 h (Pogrzeba et al. 2017).

### Analysis of elements concentration in the soil and plants samples

The bioavailable forms of Pb, Cd, and Zn in the soil were extracted using 0.01 M CaCl_2_ (for a review, see Peijnenburg et al. 2007). Extraction was conducted with 5 g of air-dried soil (< 0.25 mm) and 50 ml 0.01 M CaCl_2_ for 2 h. Bioavailable metal concentrations (Cd, Pb, Zn) were measured in filtrate using flame atomic absorption spectrometer (iCE 3500 FAAS, Thermo Scientific).

Total concentrations of Pb, Zn, Cd, Mg, Fe, and Ca in the soil (< 0.25 mm) and plant samples were obtained by flame atomic absorption spectrometry (iCE 3500 FAAS, Thermo Scientific) after microwave sample digestion in concentrated HNO_3_ and H_2_O_2_ (4:1 *v*/*v*) (ETHOS 1, Milestone, Italy).

The total nitrogen (N) concentration in soil was determined using the dry combustion method (ISO 13878:^1998^). Available phosphorus (P) and available potassium (K) concentrations in the soil were assessed according to the method described by Egner et al. (1960). Total N concentration in plant shoots and roots was measured using the titration method (Bremner 1996), whereas total phosphorus (P) and potassium (K) concentration in plant shoots and roots were estimated in previously mineralized samples using ICP (Liberty 220,Varian, USA).

### Dehydrogenase activity determination

Dehydrogenase activity was determined according to the modified method of Casida et al. (1964). Soil samples (3 g) were placed in test tubes and incubated at 25 °C for 96 h. After that, the substrate (3% *v*/*w* TTC) was added and the tubes were incubated at 25 °C for 24 h. The samples were vortexed, filtered using acetone as an extractor agent, and read at *λ* = 485 nm using UV-Vis spectrophotometer (BioPhotometer® D30, Eppendorf, Germany).

### Arbuscular mycorrhizal fungi colonization rate

For the estimation of mycorrhizal development, the roots were prepared according to a modified method of Phillips and Hayman (1970). After washing in tap and deionized water, the roots were purified in 7% KOH at 90 °C for 10 min and then rinsed in a few changes of deionized water. The root samples were acidified in 5% lactic acid for 24 h and stained with 0.01% aniline blue in lactic acid for 24 h. Subsequently, the evaluation of arbuscular mycorrhizal fungi (AMF) colonization was conducted according to Trouvelot et al. (1986) using Mycocalc software (http://www.dijon.inra.fr/mychintec/Mycocal-prg/download.html). Fifteen root fragments on one slide were observed under the microscope and rated according to the range of classes indicated in the Trouvelot et al. (1986) method. These classes give a rapid estimation of the level of mycorrhizal colonization of each root fragment and the abundance of arbuscules. The following parameters were evaluated: F%, percentage of segments showing internal colonization; M%, average percentage of colonization of root segments; A%, percentage of arbuscules in the whole root system.

### Physiological parameters

#### Plant gas exchange measurements

Plant gas exchange parameters, such as photosynthesis rate (A), stomatal conductance (*g*_s_) transpiration rate (*E*), and intracellular CO_2_ concentration (*C*_i_) were conducted on the first fully developed leaf (mostly the third from the apex). Measurements were carried out on the 18th, 32nd, and 45th day of plant cultivation in the soil using an infrared gas analyzer (LCpro+, ADC Bioscientific, UK) under controlled climate conditions (*T* = 24 °C, PAR = 1000 μmol m^−2^ s^−1^). Measurements were performed between 9 am and noon.

#### Plant pigment contents

Relative chlorophyll and anthocyanins content was measured using a plant pigment meter (DUALEX SCIENTIFIC+™, Force-A, France). The plant pigment content assay was performed immediately following the gas exchange measurements and using the same leaves as for the gas exchange measurements.

#### Chlorophyll *a* fluorescence measurements

Chlorophyll *a* fluorescence was measured using Handy Plant Efficiency Analyzer (Hansatech Instruments Ltd., UK). Before analysis, leaves were dark-adapted for 25 min using specially designed clips (LC, Hansatech Instruments, Ltd., UK). Measurements were conducted on the 45th day using the same leaves as for the plant pigment content and gas exchange measurements.

#### Plant growth measurements

At the end of the experiment, the shoot height (h) and dry biomass of plant shoots and roots were measured.

### Oxidative stress assessment

The H_2_O_2_ content was determined in leaves using the modified procedure described by Loreto and Velikova (2001) while lipid peroxidation, an indicator of oxidative cell damage, was assessed by measuring MDA content in plant leaves according to the Dhindsa et al. (1981) method.

### Statistical analysis

All data were analyzed using one-way ANOVA with LSD post hoc test (*P* < 0.05) for comparison of more than two independent groups, while Wilcoxon test (*P* < 0.05) was used for comparison of two independent groups. Principal component analyses (PCA) were performed on a correlation matrix to detect clusters of cases and correlation between investigating parameters corresponding to plant physiological status (plant growth measurements, elements concentration in shoots, gas exchange measurement, plant pigment content, mycorrhizal colonization, oxidative stress indicators). Statistical analyses were performed using Statistica v13.1 (Dell Inc., USA).

## Results

### Soil characteristics

The soil from Gliwice (HM_low_) was classified as medium loam, while from Bytom (HM_mod_) and Piekary Śląskie (HM_high_) as Silty Loam (Table 1, Table S1). The lowest soil pH was found for Bytom site, while the highest was observed in Gliwice site. Analysis of soil electrical conductivity (EC) did not show significant differences between sterilized and nonsterilized soil, demonstrating similar tendencies as pH values (Table 1). Bytom soil showed a significantly lower content of organic matter (OM) by about 1%, compared to the soil from Gliwice and Piekary Śląskie. The elemental concentration in the soils is presented in Table 1. The significantly higher N_total_ concentration (by about 20%) was found in Gliwice and Piekary Śląskie when compared to Bytom soil. No significant differences were observed in P_available_ concentration in Bytom and Gliwice. The lowest P_available_ concentration was found in Piekary Śląskie soil and it was about 63% lower in comparison with the other soils. K_available_ concentration was higher in soils from Bytom and Piekary Śląskie, where there were no significant differences, while K_available_ concentration in soil from Gliwice was nearly 50% lower. The highest Mg and Cu concentration was observed in the P_NS soil while there were no significant differences between G_NS and B_NS. G_NS soil had the highest Ca concentration (75% higher than the other soils).

**Table 1:** Soil physicochemical parameters, elements concentration, and soil microbial activity

	Experimental variants					
	G_NS	B_NS	P_NS	G_S	B_S	P_S
Physicochemical soil characteristic						
pH(H_2_O)	7.73 ± 0.01a	6.99 ± 0.04c	7.07 ± 0.01b	7.85 ± 0.01a	6.97 ± 0.01c	7.07 ± 0.03b
EC (μS cm^−1^)	174.3 ± 5.7a	83.8 ± 9.6c	105.8 ± 8.7bc	191.1 ± 13.1a	93.5 ± 12.1bc	121.3 ± 6.2b
OM (%)	5.08 ± 0.05a	4.25 ± 0.14b	5.01 ± 0.03a	5.23 ± 0.08a	4.04 ± 0.18b	5.30 ± 0.04a
Soil texture	Medium loam	Silty loam	Silty loam	Medium loam	Silty loam	Silty loam
Elements concentration in soil						
N (%)	0.19 ± 0.01a	0.16 ± 0.01b	0.20 ± 0.01a	0.2 ± 0.01a	0.15 ± 0.01b	0.19 ± 0.01a
P_available_ (mg kg^−1^)	349 ± 8a	375 ± 6a	132 ± 3c	323 ± 11b	323 ± 9b	127 ± 4c
K_available_ (mg kg^−1^)	122.3 ± 3.7c	227.7 ± 12.4ab	205.4 ± 10.5b	131.0 ± 7.3c	250.3 ± 8.3a	193.2 ± 4.8b
Mg (mg kg^−1^)	1175 ± 21b	1133 ± 57bc	1250 ± 15a	1156 ± 10bc	1119 ± 9c	1235 ± 16a
Ca (mg kg^−1^)	5686 ± 50a	3220 ± 155b	3259 ± 101b	5878 ± 108a	3105 ± 55b	3341 ± 141b
Fe (mg kg^−1^)	10,579 ± 223c	11,151 ± 332bc	12,387 ± 255a	11,542 ± 259b	9616 ± 56d	10,562 ± 208c
Cu (mg kg^−1^)	14.8 ± 0.3b	15.8 ± 0.9b	20.5 ± 1.1a	15.3 ± 0.4b	15.4 ± 0.9b	19.7 ± 1.1a
Pb (mg kg^−1^)	58.6 ± 5.3c	358.6 ± 13.4b	1603.4 ± 52.3a	59.6 ± 3.7c	327.7 ± 4.1b	1567.8 ± 31.4a
Cd (mg kg^−1^)	0.21 ± 0.09c	12.14 ± 0.30b	57.75 ± 1.86a	0.54 ± 0.19c	11.63 ± 0.15b	55.86 ± 1.04a
Zn (mg kg^−1^)	185 ± 2c	1931 ± 61b	3438 ± 199a	196 ± 10c	1820 ± 39b	3122 ± 128a
Bioavailable forms of heavy metals in soil						
Pb (mg kg^−1^)	LOQ	LOQ	LOQ	LOQ	LOQ	LOQ
Cd (mg kg^−1^)	LOQ	1.09 ± 0.06b	6.33 ± 0.02a	LOQ	1.02 ± 0.00b	5.96 ± 0.02a
Zn (mg kg^−1^)	0.23 ± 0.05c	47.94 ± 0.78b	103.15 ± 2.26a	0.16 ± 0.02c	41.94 ± 1.66b	101.73 ± 0.61a
Soil microbial activity						
DHA (μg TPF g^−1^ d.w.)	581.56 ± 30.32a	83.78 ± 3.92b	73.39 ± 10.47b	19.39 ± 2.19c	11.25 ± 0.25c	8.79 ± 1.00c

Values are means ± SE (*n* = 3). Lower case letters (a, b, c) denote significant difference between means in a row according to Fisher LSD test at *P* ≤ 0.05

*G* soil from Gliwice site (HM_low_), *B* soil from Bytom site (HM_mod_), *P* soil from Piekary Śląskie site(HM_high_), *NS* nonsterile soil, *S* sterile soil, *EC* electrical conductivity, *OM* organic matter, *DHA* dehydrogenase activity, *LOQ* limit of quantification

Concentration of bioavailable forms of the investigated heavy metals, Pb in each variant and Cd in control variants (G_NS and G_S) were below detection limit. The highest bioavailable Zn and Cd concentration was found in Piekary Śląskie soil, while moderate and the lowest were found in Bytom and Gliwice soil respectively. Sterilization did not affect soil elemental composition except for P_available_ concentration. It was found that sterilization of soil from Gliwice and Bytom resulted in a decrease of P_available_ concentration in those experimental variants.

Dehydrogenase activity (DHA), measured at the end of the experiment, was very much lower in the sterilized soils compared to nonsterilized which showed that sterilization was successful (Table 1). Sterilization decreased DHA in noncontaminated G_S soil by 96.7%, whereas in soils contaminated by heavy metals DHA, it was diminished by 87–88%. In nonsterilized soil, the highest DHA was found for noncontaminated soil (G_NS), while the lowest for the most contaminated soil (P_NS).

### Concentration of elements in shoots and roots

The concentrations of investigated elements in plants are presented in Table 2. Concentrations of elements in root and shoots, in most cases, correspond to the values obtained during soil elemental analysis; however, the most notable differences were driven by comparisons between plants cultivated in sterilized or nonsterilized soils.

**Table 2:** Concentration of elements in roots and shoots and plant growth parameters of *Zea mays*

		Experimental variants					
		G_NS	B_NS	P_NS	G_S	B_S	P_S
N (%)	root	0.95 ± 0.03abc	0.98 ± 0.05a	0.85 ± 0.03bc	0.83 ± 0.06 cd	0.77 ± 0.04c	0.97 ± 0.02ab
	shoot	1.20 ± 0.04ab	0.98 ± 0.04 cd	0.90 ± 0.02d	1.08 ± 0.07bc	0.85 ± 0.03d	1.23 ± 0.06a
P (mg kg^−1^)	root	1497 ± 77b	1978 ± 19a	678 ± 74c	1218 ± 270b	2068 ± 184a	589 ± 109c
	shoot	3212 ± 81a	3137 ± 64a	1041 ± 70b	3398 ± 89a	3433 ± 99a	1007 ± 34b
K (mg kg^−1^)	root	4651 ± 699d	20,363 ± 692a	11,399 ± 614c	3397 ± 880d	15,923 ± 1060b	9722 ± 613c
	shoot	18,556 ± 1299d	39,391 ± 716a	29,309 ± 1007b	9946 ± 1132e	33,006 ± 1583b	24,216 ± 390c
Mg (mg kg^−1^)	root	2774 ± 341a	1923 ± 90b	1465 ± 141bc	1748 ± 156b	1189 ± 137c	1422 ± 80bc
	shoot	3178 ± 315a	1356 ± 66 cd	1593 ± 55bc	2840 ± 158a	1106 ± 44d	1810 ± 53b
Ca (mg kg^−1^)	root	4723 ± 100a	3737 ± 195b	3548 ± 154b	4398 ± 86a	3497 ± 188b	3670 ± 474b
	shoot	5064 ± 123a	4468 ± 123b	4232 ± 123b	4268 ± 98b	3765 ± 111c	3809 ± 118c
Fe (mg kg^−1^)	root	2785 ± 625a	2103 ± 406ab	838 ± 202d	1827 ± 71ab	1684 ± 74b	803 ± 188d
	shoot	51.50 ± 5.97bc	91.43 ± 17.62a	65.55 ± 12.74abc	72.18 ± 8.61ab	82.60 ± 20.13ab	40.87 ± 3.95c
Cu (mg kg^−1^)	root	15.41 ± 4.80a	11.00 ± 1.67a	5.48 ± 1.06a	11.70 ± 3.37a	5.46 ± 1.22a	6.97 ± 0.60a
	shoot	2.25 ± 0.29a	2.19 ± 0.88a	1.34 ± 0.39a	2.37 ± 0.58a	2.25 ± 0.52a	1.88 ± 0.38a
Pb (mg kg^−1^)	root	42.23 ± 5.24b	64.65 ± 10.70b	125.03 ± 23.38a	32.39 ± 4.87b	64.96 ± 5.30b	169.41 ± 35.67a
	shoot	9.24 ± 1.62b	13.82 ± 3.74b	20.30 ± 3.29a	13.19 ± 1.96b	8.78 ± 2.19b	10.40 ± 1.25b
Cd (mg kg^−1^)	root	2.17 ± 0.49c	47.15 ± 9.36b	81.24 ± 7.68a	2.13 ± 0.69c	27.12 ± 9.79b	76.98 ± 11.94a
	shoot	0.55 ± 0.02c	0.83 ± 0.27c	2.40 ± 0.50b	0.22 ± 0.12c	1.82 ± 0.49b	7.86 ± 0.78a
Zn (mg kg^−1^)	root	246.5 ± 30.6c	1222.3 ± 84.2b	1692.3 ± 167.9a	175.8 ± 27.2c	950.0 ± 138.1b	1841.7 ± 195.9a
	shoot	72.94 ± 20.01d	300.03 ± 27.84c	568.81 ± 42.39b	61.48 ± 14.17d	299.79 ± 41.14c	959.93 ± 58.61a
Growth parameters							
H (cm)		59 ± 1c	61 ± 1c	61 ± 1c	81 ± 3a	73 ± 2b	67 ± 3bc
root d.w. (g)		0.58 ± 0.03c	1.46 ± 0.12ab	0.65 ± 0.10c	1.59 ± 0.34ab	1.77 ± 0.33a	1.02 ± 0.35bc
shoot d.w. (g)		2.78 ± 0.44c	4.29 ± 0.38c	2.86 ± 0.22c	9.34 ± 1.38a	6.83 ± 0.54b	4.52 ± 0.46c

Values are means ± SE (*n* = 4). Lower case letters (a, b, c, d) denote significant difference between means in a row according to Fisher LSD test at *P* ≤ 0.05

*G* soil from Gliwice site (HM_low_), *B* soil from Bytom site (HM_mod_), *P* soil from Piekary Śląskie site(HM_high_), *NS* nonsterile soil, *S* sterile soil, *H* shoot height

There were significant differences in N, K, and Mg root concentration, with a decrease of these elements concentration in roots of plants cultivated on B_S soil when compared to its nonsterilized counterpart. In addition, a decrease in roots Mg concentration by about 37% was found for G_S soil in comparison to its nonsterilized counterpart.

Nitrogen concentration in shoots was significantly lower (10%) for plants cultivated in G_S soil and significantly higher (37%) for plants cultivated in P_S soil as compared to their nonsterilized counterparts. Significantly, lower values for K shoot concentration were found in variants with sterilized soil. Interestingly, Ca concentration in shoots was significantly lower in sterilized soil, despite the fact that differences between concentrations of this element in roots were statistically insignificant. Sterility of soil significantly affected Cd concentration in shoots; it caused higher concentrations in plants cultivated in B_S and P_S soil by about 54 and 70% when compared to plants cultivated in B_NS and P_NS soil, respectively. A similar relationship was found for Zn shoot concentration; however, this was only seen in the Piekary Śląskie (HM_high_) soil.

### Mycorrhizal parameters

**Fig. 1:**
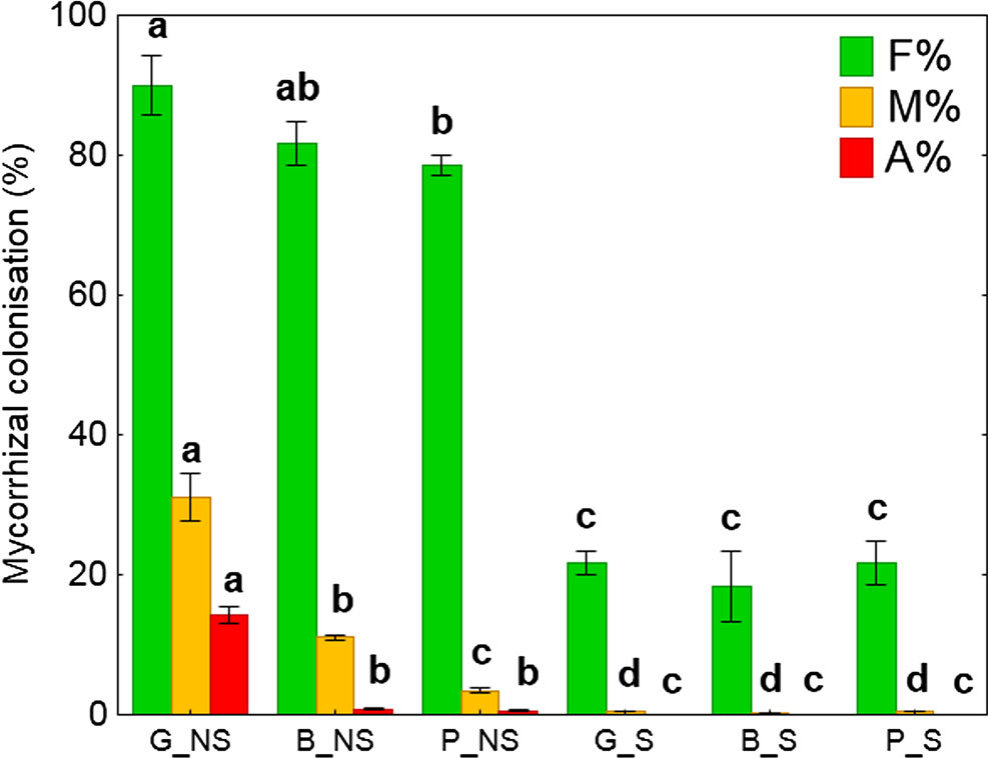
Degree of arbuscular mycorrhizal fungi colonization of roots and arbuscules formation. Values are means ± SE. (*n* = 4). Lower case letters (a, b, c) denote significant difference between means according to Fisher LSD test at *P* ≤ 0.05. G, soil from Gliwice site (HM_low_); B, soil from Bytom site (HM_mod_); P, soil from Piekary Śląskie site (HM_high_); NS, nonsterilized soil; S, sterilized soil; F%, percentage of segments showing internal colonization; M%, average percentage of colonization of root segments; A%, percentage of arbuscules in the whole root system

Mycorrhizae colonization levels were significantly affected by HM concentration in soil (Fig. 1). The lowest mycorrhizal intensity (M%) was found in P_NS and B_NS plant roots which were lower by 86 and 70% in comparison G_NS, respectively. G_S, B_S, and P_S plant roots were devoid of AMF colonization. Moreover, in contrast to P_NS (HM_high_) and B_NS (HM_mod_) plants, G_NS (HM_low_) showed the *Arum-*type mycorrhizal colonization. In plants grown on the HMC soils, AMF, when present, exhibited Paris-type mycorrhizal colonization (Fig. S2).

Dark septate endophytes (DSE) were found in plants cultivated on HM-contaminated soils (P_NS and B_NS), while they were absent in root samples from G_NS. The mycelium was brownish and occurred mainly in root fragments where arbuscules were not present. Both dematiaceous septate hyphae and microsclerotia were observed (Fig. S3).

### Chlorophyll and anthocyanins content

**Fig. 2:**
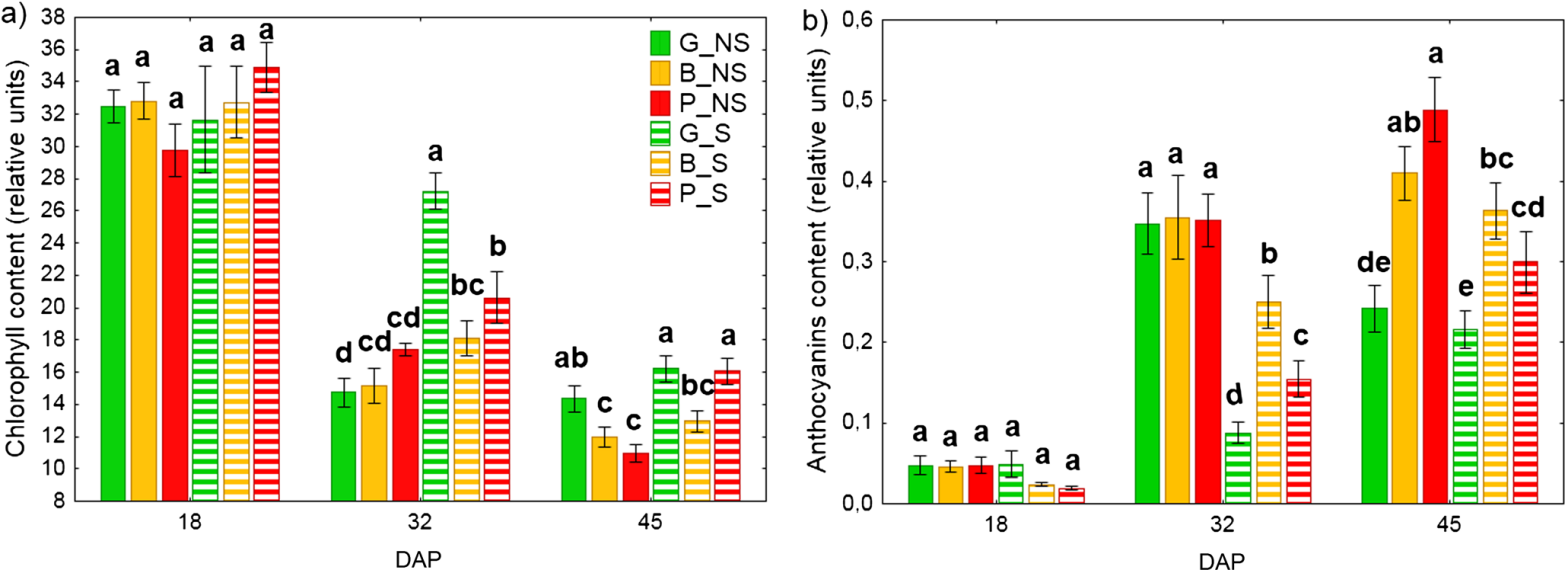
Effect of autochthonous microorganisms on chlorophyll (**a**), flavonoids (**b**) and anthocyanins (**c**) leaves content. Values are means ± SE, (*n* = 16). Lower case letters (a, b, c) denote significant difference between means according to Fisher LSD test at *P* ≤ 0.05. A statistical analysis was performed for each day separately. G, soil from Gliwice site (HM_low_); B, soil from Bytom site (HM_mod_); P, soil from Piekary Śląskie site (HM_high_); NS, nonsterilized soil; S, sterilized soil; DAP, day after planting

Relative chlorophyll content in *Z. mays* leaves decreased during the experiment, irrespectively of the studied experimental treatment (Fig. 2a). No significant difference in relative chlorophyll content was observed on the 18th day from planting across all experimental variants. The largest decrease of relative chlorophyll content was observed on the 45th day after planting. Among plants cultivated on nonsterilized soil, the greatest decrease of relative chlorophyll content was found in B_NS (HM_mod_) and P_NS (HM_high_) plants, which were 63% lower relative to that measured on the 18th day. In addition, by the 45th day, G_NS (HM_low_) plants had significantly higher relative chlorophyll content than plants cultivated on the other nonsterilized soils. In contrast to measurements taken on the 18th day, significant differences were seen in relative chlorophyll content in leaves on the subsequent measuring dates (32nd and 45th days) with chlorophyll tending to be significantly higher in plants cultivated on sterilized soils compared to nonsterilized ones. Among plants cultivated on sterilized soil, by the 32nd and 45th days, the highest relative chlorophyll content was found in G_S and P_S plants, with no significant differences noted between them, while the lowest relative chlorophyll content was found for B_S plants.

Anthocyanin levels also increased during cultivation (Fig. 2b). Analysis carried out on the 18th day sampling showed no significant differences between any of the treatments. By the 32nd day, a large increase in anthocyanin index was observed across all soils, about 64% higher than earlier measurements in plants cultivated on the nonsterilized soils. Leaves of plants grown on sterilized soils also showed an increased anthocyanin index, but the values were lower in comparison with their nonsterilized counterparts by 74%, 29%, and 55% for G_S, B_S, and P_S plants respectively. This 32nd day sampling revealed significant differences between sterilized and nonsterilized soils. Within the HM status groups, nonsterilized soils were not different to each other; in contrast, all three soil types within the sterilized group were significantly different to each other at this time point.

With the exception of G_NS, all treatments had again increased in anthocyanin index by the 45th day sampling. Among plants cultivated on nonsterilized soil, the highest values were found in leaves of P_NS plants, which was ten times higher than that of the first (18th day) measurements. The lowest values were found in leaves of G_NS. Among plants cultivated on sterilized soil, the highest values were found in leaves of B_S and P_S plants. Across all treatments, the lowest anthocyanin index tended to be found on the uncontaminated sterilized and unsterilized soils.

### Chlorophyll *a* fluorescence

#### Chlorophyll *a* fluorescence induction curve

The transient sector between bands (O, J, I, and P) of the chlorophyll induction curve provides important information about the kinetic energy transfer and conversion between the different components of photosynthetic apparatus related to the light-dependent phase of photosynthesis (for review, see Goltsev et al. 2016; Kalaji et al. 2014; Strasser et al. 2004).

**Fig. 3:**
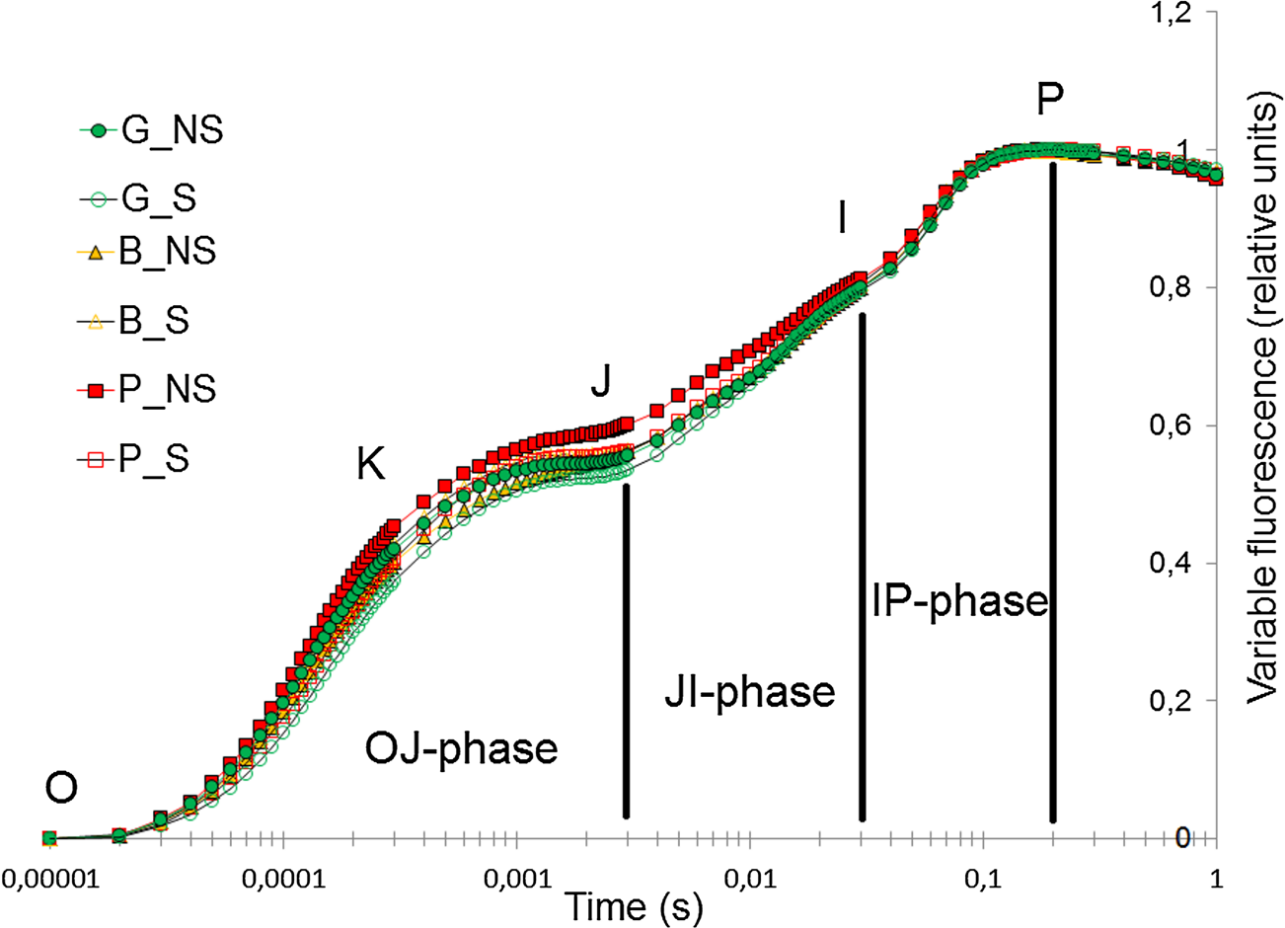
Relative chlorophyll *a* fluorescence induction curve. Values are means ± SE. (*n* = 16). G, soil from Gliwice site (HM_low_); B, soil from Bytom site (HM_mod_); P, soil from Piekary Śląskie site (HM_high_); NS, nonsterilized soil; S, sterilized soil; 0, minimal fluorescence intensity (F_O_); K, band at 0.0003 s; J, band at 0.002 s; I, band at 0.002 s; P, maximal fluorescence intensity (F_M_)

Across all experimental treatments, the only differences in the fluorescence transient curve were found at K- and J-band (Fig. 3). No differences were found at I-band. Different doses of HM in soil as well as soil sterility did not influence I-P phase.

#### Chlorophyll *a* fluorescence parameters

The significant differences in most of JIP-test parameters (Table 3) were visible between sterilized and nonsterilized variants of the same soil, as well as between variants of different concentration of heavy metals in soil. Significantly, higher values of minimal fluorescence intensity (F_O_), maximal fluorescence intensity (F_M_), maximum quantum yield of primary photochemical reactions (ϕPo – F_V_/F_M_), indicator of photosystem II (PSII) functional activity normalized to the absorbed energy (PI_ABS_) and total performance index; indicating the integral functional activity of PSII, photosystem I (PSI), and intersystem electron transport chain (PI_total_) found in *Z. mays* leaves cultivated in soil from Gliwice (HM_low_) and Piekary Śląskie (HM_high_). However, no significant differences were found between these parameters for plants cultivated in the Bytom (HM_mod_) sterilized and nonsterilized soil. In contrast, a significant decrease of absorption energy flux per active reaction centers (ABS/RC) and dissipated energy flux per RC (DIo/RC) was found in the same experimental variants, containing soil from Gliwice (HM_low)_ and Piekary Śląskie. Interestingly, ABS/RC, DIo/RC, PI_ABS_, and PI_total_ in the nonsterilized soil did not show any differences between Gliwice (HM_low)_ and Bytom (HM_mod_) (simultaneously with lower values for Piekary Śląskie (HM_high_)). On sterilized soil significantly higher values were found for plants cultivated in Gliwice (HM_low)_ soil as compared to the B_S and P_S and no significant differences were found between the variants containing excessive level of heavy metals. Regarding differences in F_O_ values between nonsterilized experimental variants, no significant differences were found between B_NS and other nonsterilized variants, while plants grown on Gliwice (HM_low_) soil showed 25% higher values when compared to P_NS. However, on sterilized variants, the highest Fo was found for P_S, while 10 and 34% lower values were found for G_S and B_S respectively. Similar pattern was seen for F_M_; however, B_NS differ significantly from other nonsterilized variants, and on sterilized variants, no significant differences were found between G_S and P_S. The most common JIP-test parameter, ϕPo, also known as F_V_/F_M_, showed the same pattern of changes in the sterilized and nonsterilized soil. No significant differences were found between plants cultivated in soil from Gliwice (HM_low_) and Bytom (HM_mod_), while significantly lower values were found for plants cultivated on soil from Piekary Śląskie (HM_high_).

**Table 3:** JIP-test parameters of *Zea mays*

	Fo	Fm	ABS/RC	DIo/RC	TRo/RC	ETo/RC	ϕPo	ϕEo	PI_ABS_	PI_total_
G_NS	369 ± 21bc	1268 ± 75bc	3.74 ± 0.08b	1.12 ± 0.05b	2.62 ± 0.04a	1.11 ± 0.04a	0.70 ± 0.01bc	0.30 ± 0.01ab	0.51 ± 0.06b	0.42 ± 0.04b
G_S	406 ± 14b*	1572 ± 73a*	3.30 ± 0,09c*	0.86 ± 0.04c*	2.44 ± 0.07a^ns^	1.09 ± 0.02a^ns^	0.74 ± 0.01a*	0.34 ± 0.02a^ns^	0.77 ± 0.07a*	0.60 ± 0.06a*
B_NS	330 ± 18 cd	1079 ± 73d	3.62 ± 0.09b	1.14 ± 0.06b	2.48 ± 0.04a	1.10 ± 0.03a	0.69 ± 0.01c	0.31 ± 0.01ab	0.52 ± 0.05b	0.42 ± 0.04b
B_S	333 ± 13cd^ns^	1191 ± 53bcd^ns^	3.62 ± 0.08b^ns^	1.02 ± 0.04b	2.59 ± 0.04a^ns^	1.12 ± 0.04a^ns^	0.72 ± 0.01ab*	0.31 ± 0.01ab^ns^	0.57 ± 0.05b^ns^	0.44 ± 0.03b^ns^
P_NS	296 ± 19d	895 ± 79e	3.98 ± 0.09a	1.4 ± 0.08a	2.58 ± 0.6a	1.02 ± 0.06a	0.65 ± 0.01d	0.26 ± 0.02c	0.36 ± 0.05c	0.30 ± 0.04c
P_S	448 ± 12a*	1435 ± 59ab*	3.62 ± 0.09b*	1.16 ± 0.07b*	2.46 ± 0.5a^ns^	1.02 ± 0.05a^ns^	0.68 ± 0.01 cd*	0.28 ± 0.02bc^ns^	0.46 ± 0.05b*	0.40 ± 0.05b*

Values are means ± SE. (*n* = 16). Lower case letters (a, b, c) denote significant differences between experimental variants according to Fisher LSD test at *P* ≤ 0.05, while asterisks (*) denote significant difference between sterilized and nonsterilized variants for each soil separately according to Mann-Whitney *U* test at *P* ≤ 0.05

*G* soil from Gliwice site (HM_low_), *B* soil from Bytom site (HM_mod_), *P* soil from Piekary Śląskie site (HM_high_), *NS* nonsterilized soil, *S* sterilized soil, *Fo* minimal fluorescence intensity, *F*_*M*_ maximal fluorescence intensity, *ABS/RC* absorption energy flux per active RC, *DIo/RC* dissipated energy flux per RC (at *t* = 0), *TRo/RC* flux of excitation energy trapped per active RC (at *t* = 0), *ETo/RC* electron transport flux (further than Q_A_-) per RC (at *t* = 0), *ϕPo* maximum quantum yield of primary photochemical reactions (at *t* = 0), *ϕEo* quantum efficiency of electron transfer from Q_A_—to electron transport chain beyond Q_A_—(at *t* = 0), *PI*_*ABS*_ performance index, an indicator of PSII functional activity normalized to the absorbed energy, *PI*_*total*_ total performance index, indicating the integral functional activity of PSII, PSI, and intersystem electron transport chain

### Gas exchange parameters

**Fig. 4:**
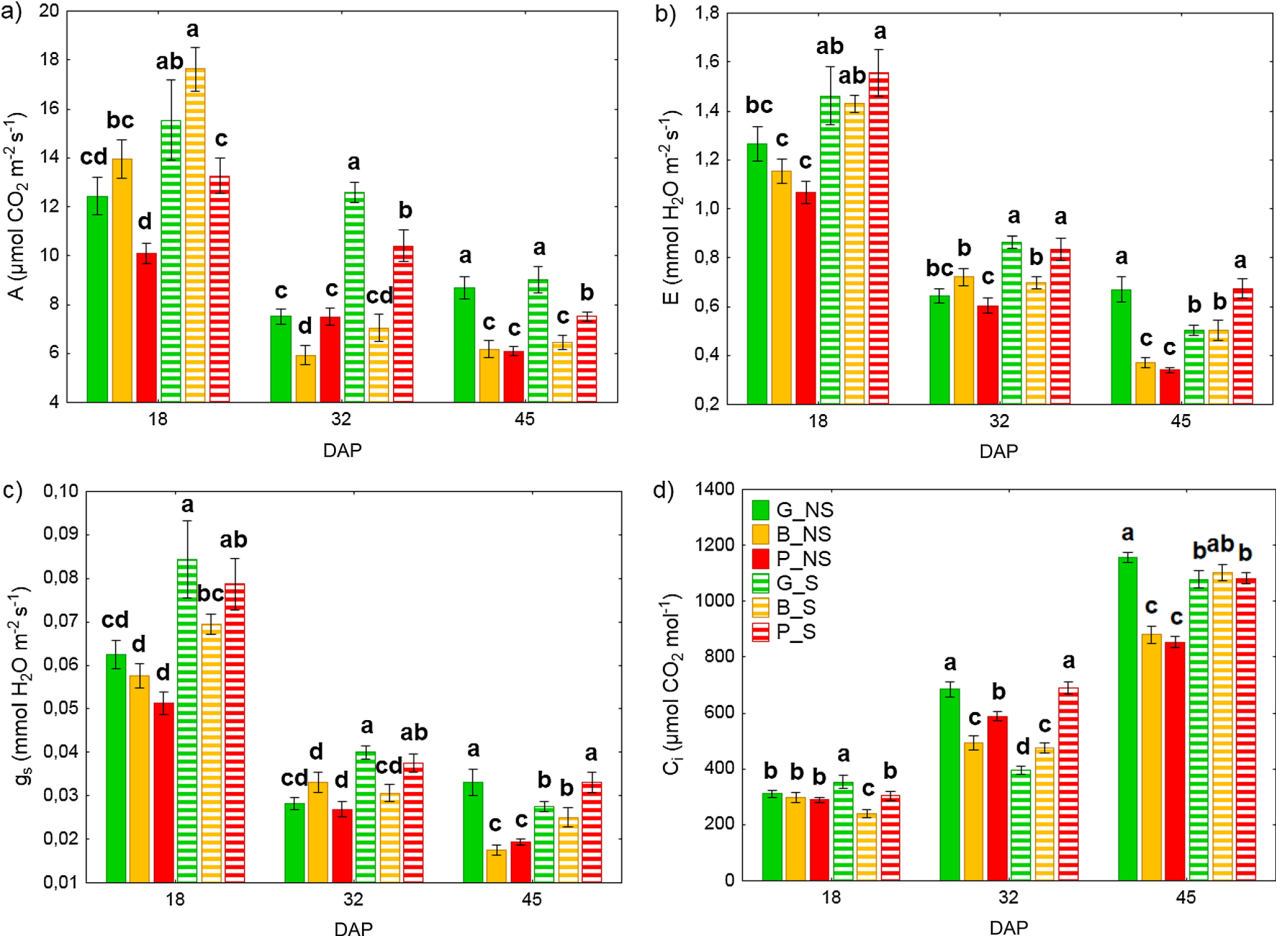
Effect of autochthonous microorganisms on gas exchange parameters in maize plants. Values are means ± SE (*n* = 16). Lower case letters (a, b, c) denote significant difference between means according to Fisher LSD test at *P* ≤ 0.05. A statistical analysis was performed for each day separately. G, soil from Gliwice site (HM_low_); B, soil from Bytom site (HM_mod_); P, soil from Piekary Śląskie site (HM_high_); NS, nonsterilized soil; S, sterilized soil; DAP, day after planting; A, photosynthesis rate (a); E, transpiration rate (b); gs, stomatal conductance (c); ci, intracellular CO2 concentration (d)

Measurements taken on the 18th day after planting showed significant differences in photosynthetic rate (A) between plants grown on nonsterilized and sterilized soils (Fig. 4a). Plants from sterilized soils showed a higher photosynthetic rate in comparison with those of nonsterilized soils, by 25%, 26%, and 31% for G_S (HM_low_), B_S (HM_mod_) and P_S (HM_high_) plants, respectively. Among the plants cultivated on sterilized soils, the highest rate was found for B_S plants and the lowest for P_S plants. Plants cultivated on moderately HM-contaminated (HM_mod_) and -sterilized soil (B_S) did not differ significantly from their uncontaminated control (HM_low_) (G_S); results were similar for plants grown on the sterilized soils. Analysis carried out on the 32nd day showed a decreasing trend in photosynthetic rate; here, the highest rate was observed for G_S plants with this treatment showing the smallest decrease between the 18th and 32nd day of sampling (18%). There were no significant differences between G_NS and P_NS plants on the 32nd day. The lowest photosynthetic rate was observed for both B_NS and B_S (57 and 60%, respectively). After the 45th day of cultivation the highest rate was noted for the uncontaminated G_NS and G_S plants with no significant difference between the two soil sterilization treatments. Across all soils (sterilized and nonsterilized) plants from the HM-contaminated treatments tended to have lower photosynthetic rates than those from the uncontaminated one. However, values obtained for plants cultivated on highly contaminated sterilized soil were significantly higher when compared to the other values obtained for plants grown on contaminated soils.

Transpiration rate (*E*) and stomatal conductance (*g*_s_) showed a similar declining trend over time (Fig. 4b, c). Measurements taken on the 18th day showed significant differences between plants grown on nonsterilized and sterilized soils. Plants from nonsterilized soils showed a lower transpiration rate in comparison with these of sterilized soils by 13%, 19%, and 31% for G_NS, B_NS, and P_NS plants, respectively. Among plants cultivated on sterilized soils, the highest rate was found for P_S plants and the lowest for B_S plants. For plants cultivated on nonsterilized soil, *E* value was the highest in G_NS plants and the lowest in P_NS plants. The highest differences in *E* and *g*_*s*_ values were found between the 18th and 45th day after planting—by about 60% for all variants.

Intracellular concentration of CO_2_ (*C*_i_) increased over time (Fig. 4d). Analysis carried out on the 18th day sampling showed no significant differences within the HM treatments in the nonsterilized soils but did show differences between all three HM levels in the sterilized soils. Among these plants cultivated on sterilized soils, the highest rate was found for G_S (HM_low_) plants and the lowest for B_S plants. After 32 days, a large increase in intracellular concentration of CO_2_ was seen compared to values recorded on the 18th day, particularly in G_NS plants (by 120%). In other variants, there was also high increase of C_i_ value by 65%, 98%, 103%, and 127%, for B_NS, B_S, P_NS, and P_S plants, respectively. On the 45th day, C_i_ values were generally significantly higher on the sterilized soils though the highest value here individually was seen on the nonsterilized G_NS soil. Furthermore, among plants cultivated on nonsterilized soil there were no significant differences between the two HM contamination levels B_NS and P_NS, which recorded the lowest intracellular concentration of CO_2_. Among plants cultivated on sterilized soils, there were no significant differences in C_i_ concentrations between the different HMC levels.

### Plant growth parameters

#### Shoot length

Plants grown on sterilized soils had longer shoots than those on nonsterilized soils, with the exception of P_NS and P_S, where differences in plant height were insignificant (Table 2). Among plants cultivated on sterilized soils, the highest shoot length was noted in G_S (81 ± 3 cm) plants, and the lowest in P_S plants, both were significantly different from B_S (73 ± 2 cm) plants. Among plants cultivated on nonsterilized soils, no significant differences between treatments were found.

#### Root and shoot biomass

Shoot biomass was generally lower for plants cultivated on nonsterilized soil in comparison with those cultivated on sterilized ones by 63%, 17%, and 36% for G_NS, B_NS, and P_NS, respectively (Table 2). Similar trends were observed for root biomass, which was lower in plants cultivated on nonsterilized soil when compared to plant cultivated on sterilized soil by 70%, 37%, and 63% for G_NS, B_NS, and P_NS experimental variants respectively. Among plants from nonsterilized soils, the highest root and shoot biomass was found for plant cultivated in Bytom soil (HM_mod_); however, on sterilized soil, the highest root biomass was found for plant cultivated on G_S and B_S soil while the highest shoot biomass was found for plants cultivated on G_S soil.

### Oxidative stress assessment

#### H_2_O_2_ content in plant leaves

**Fig. 5:**
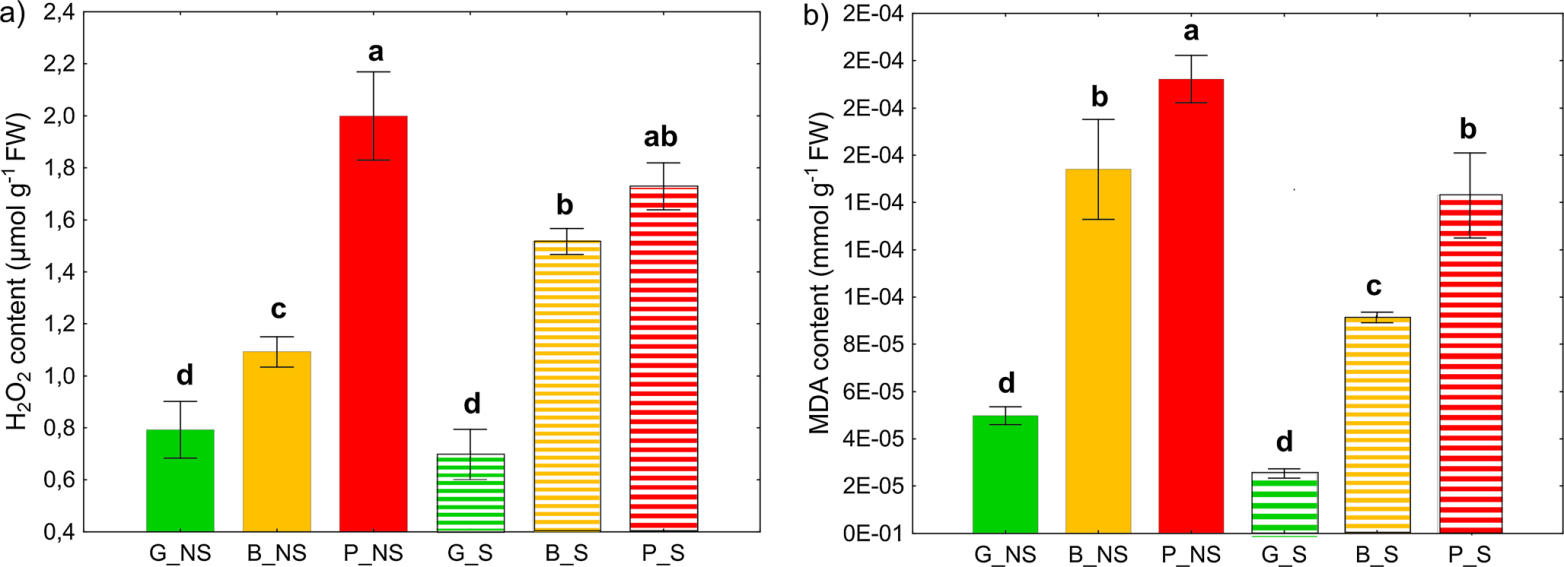
Effect of autochthonous microorganisms on MDA (**a**) and H_2_O_2_ (**b**) leaves content. Values are means ± SE. (*n* = 4). Lower case letters (a, b, c) denote significant difference between means according to Fisher LSD test at *P* ≤ 0.05. G, soil from Gliwice site (HM_low_); B, soil from Bytom site (HM_mod_); P, soil from Piekary Śląskie site (HM_high_); NS, nonsterilized soil; S, sterilized soil

H_2_O_2_ content in the leaves was generally lower for plants grown in sterilized soils compared to nonsterilized soils though for B_S specifically the opposite was true (Fig. 5a). Among plants cultivated on nonsterilized and sterilized soil, the highest content of H_2_O_2_ was found on the HM_high_ soils (P_NS and P_S), while the lowest content of H_2_O_2_ was found in the HM_low_ controls (G_NS and G_S). Maize leaves from contaminated soils typically showed H_2_O_2_ levels twice as high as their corresponding controls. Between the soil sterilization treatments there were no significant differences with the exception of HM_mod_, where the sterilized treatment did show a significantly higher H_2_O_2_ value compared to the nonsterilized.

#### MDA content in plant leaves

Similarly to the H_2_O_2_ results, MDA content in the leaves was lower in plants cultivated on sterilized soils compared to nonsterilized soils. Again, as heavy metal contamination in the soil increased, the MDA content in the leaves of both groups increased correspondingly (Fig. 5b).

### Principal component analysis (PCA)

**Fig. 6:**
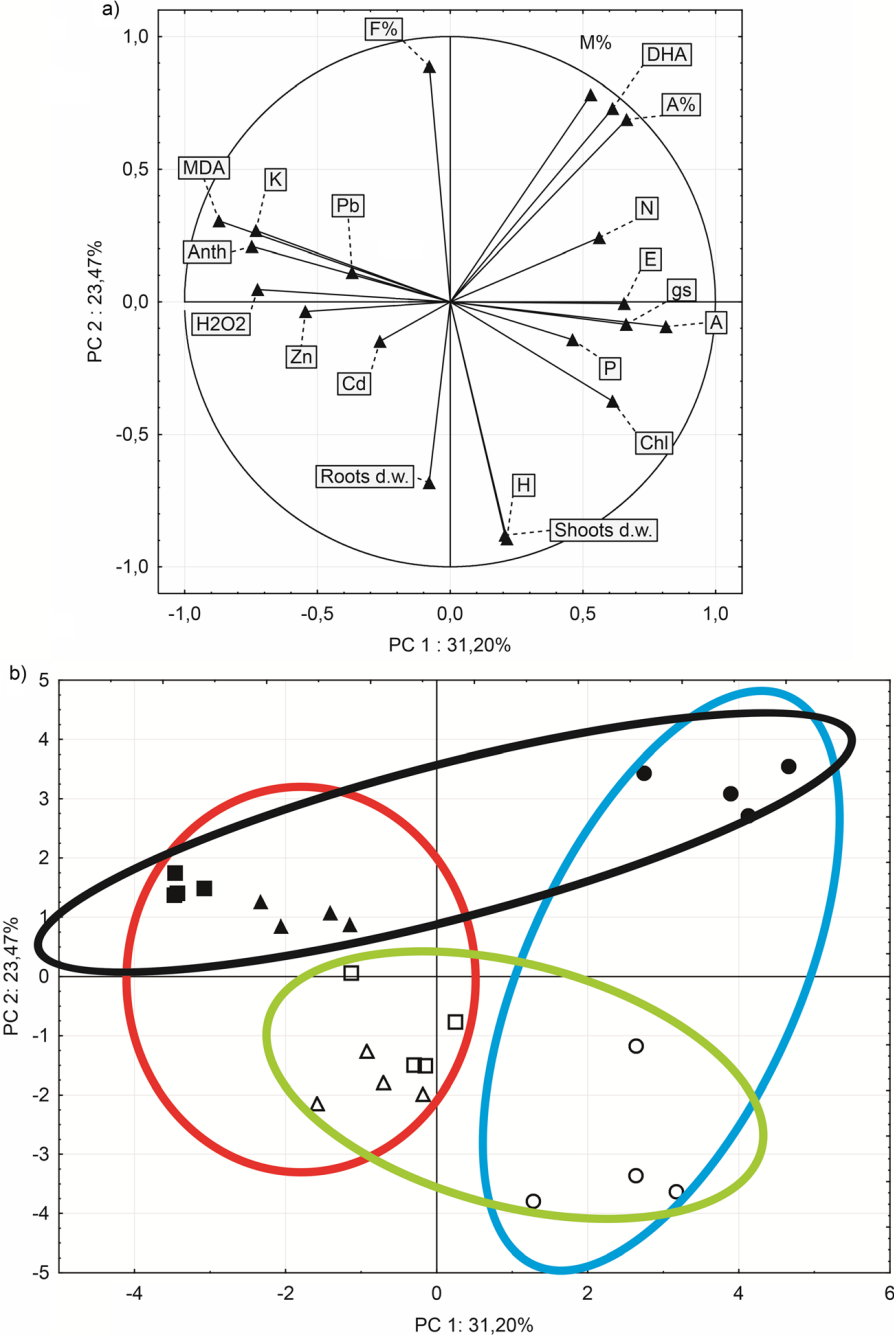
Principal component analysis (PCA) distinguished into two parts: **a** correlation between variables along two PCA axis (PC1 × PC2) and **b** ordination of case along two PCA axis (PC1 × PC2). PCA was performed on correlation matrix. Closed figures correspond to plant cultivated in nonsterilized soil, while open figures corresponds to plant cultivated in sterilized soil. A white circle indicates a plant cultivated in soil from Gliwice site. A white triangle indicates plants cultivated in soil from Bytom site, a white square indicates plants cultivated in soil from Piekary Śląskie site, a blue ellipse indicates cases corresponding to plant cultivated on uncontaminated soil, a red ellipse indicates cases corresponding to plants cultivated on two contaminated soils, a green ellipse indicates cases corresponding to plants cultivated on sterilized soil, a black ellipse indicates cases corresponding to plants cultivated on nonsterilized soil, F% indicates percentage of segments showing internal colonization, M% indicates average percentage of colonization of root segments, A% indicates percentage of arbuscules in the whole root system. DHA, dehydrogenase activity; A, photosynthesis rate; E, transpiration rate; gs, stomatal conductance; Chl, leaves chlorophyll content; Anth, leaves anthocyanins content; N, shoot N concentration; P, shoot P concentration; K, shoot K concentration; Cd, shoot Cd concentration; Pb, shoot Pb concentration; Zn, shoot Zn concentration; MDA, leaves MDA concentration; H2O2, leaves H_2_O_2_ concentration; H, shoot height

Principal component analysis (Fig. 6a, b) was performed on correlation matrix of all parameters to detect relationships between those corresponding to the efficiency of the photosynthetic apparatus (photosynthesis rate, plant pigments content), plant growth parameters, HM concentration in plant shoots (Pb, Cd, Zn), primary mineral macronutrients concentration in shoots (N, P, K), oxidative stress indicators in plant leaves (H_2_O_2_, MDA), and microbiological indicators (F%, M%, A%, DHA). The first principal component (PC1) is conditioned mostly by parameters of photosynthetic apparatus efficiency, element concentrations, and oxidative stress indicators, while the second principal component (PC2) is conditioned by plant growth parameters and microbial indicators (Fig. 6a). Parameters included in the PCA were assigned to four different correlated parameter groups. The first group contains microbial indicators (F%, M%, DHA, A%), the second group contains gas exchange measurements (*A*, *g*_*s*_, *E*), chlorophyll content in leaves (Chl) and two primary mineral macronutrients concentration in shoots (N and P). The third group consists of plant growth parameters with the last group consisting of HMs concentration in shoots (Pb, Cd, Zn), K concentration in shoots, anthocyanins, and oxidative stress indicators (MDA and H_2_O_2_).

According to their location in a coordination system (Fig. 6b) based on the two principal components (PC1 × PC2), it is possible to distinguish four clusters. The first two clusters distinguish between plants cultivated on HM-contaminated and -uncontaminated soils. The next two distinguish between nonsterilized and sterilized soil. It can be seen in Fig. 6b that the distance between cases corresponding to plants cultivated on sterilized and nonsterilized contaminated soils is shorter when compared to distance between cases corresponding to plants cultivated on sterilized and nonsterilized uncontaminated soil. A similar result was also seen for distance between sterilized contaminated and uncontaminated variants, which was shorter when compared to distance between nonsterilized contaminated and uncontaminated variants.

## Discussion

In general, *Z. mays* plants were characterized by better growth and physiological status on sterilized soil as compared to the nonsterilized soil. Bowen and Rovira (1961) obtained similar results for root development on sterilized and nonsterilized soils for *Solanum lycopersicon* Mill., *Trifolium* sp. and *Phalaris arundinacea* L., suggesting that the presence of microorganisms reduced the primary root growth in those plants. This phenomenon could be associated with competition between plant roots and rhizosphere microorganisms for mineral N and other mineral nutrients. At the initial stage of plant development microorganisms outcompete plant roots for mineral nutrients, because of rapid growth rates and high surface-area-to-volume ratios compared with those of root hairs. However, over longer periods of cultivation microorganisms cannot compete so effectively with plants for nutrients (Kuzyakov and Xu, 2013). Another possible explanation for better growth is that nutrients could be released into soils from dead microorganisms following soil sterilization (Passioura 2002). To address this possibility, an elemental analysis was carried out on both sterilized and nonsterilized soils assuming that there is no significant fertilization effect of the sterilization.

The results obtained for nonsterilized soil indicated that with an increase of heavy metal (HM) contamination, a reduction in dehydrogenase activity (DHA) occurs. However, the differences between values obtained for the two contaminated soils were statistically insignificant. Chen et al. (2014) reported that long-term exposure of microorganisms to heavy-metal pollution results in a decrease of microbial biomass and corresponding activity as well as a reduction of microbial community diversity. This suggestion is in agreement with our observation of significantly lower arbuscular bycorrhiza fungi (AMF) root colonization and DHA on heavy metal-contaminated (HMC) soils as compared to the uncontaminated soil. Previous studies from Zn- and Pb-mining regions showed a strong negative effect of soil HM contamination on AMF abundance and diversity (Zarei et al. 2010). In addition, our results suggest that AMF on the contaminated soils displayed Paris-type mycorrhiza, while on uncontaminated soil AMF followed the Arum type, agreeing with results reported by Vogel-Mikuš et al. (2005). Microscopic root observation in our study also indicated the presence of DSE and microsclerotia on both contaminated soils, while there were no visible such structures on samples from uncontaminated soil. Zhang et al. (2013) reported that in most plants grown on lead and zinc slag heaps the colonization of roots by DSE appeared. In addition, presence of DSE in Piekary Śląskie (HM_high_) soil was previously reported by Gucwa-Przepióra et al. (2013). Those endophytes may be integral to the function of HM-contaminated ecosystems due to their function as replacements or complements of mycorrhizae damaged by pollutants (Zhang et al. 2013). Despite successful sterilization of soil, low DHA was observed for these sterilized soils which could be caused by secondary soil contamination by deposition of microbes from the air (Despres et al. 2012).

Besides soil specificity, there were almost no differences in mineral macronutrient concentration in shoots between nonsterilized and sterilized variants, except K and Ca. The lower K and Ca concentrations in shoots were observed on sterilized soils as compared to nonsterilized soils. However, due to higher biomass production on sterilized soils, competition between plants and microorganisms for mineral nutrients cannot be excluded. The phenomenon of competition between plants and microorganisms for N was previously reviewed by Kuzyakov and Xu (2013). In addition, it was found that on HM_high_-sterilized soil (P_S), there were considerably higher shoot N concentrations when compared to the nonsterilized equivalent (P_NS). Although lower K and Ca concentration in plant shoots grown on sterilized soil could be associated with higher Cd shoot concentrations in plants cultivated on HMC soils (Ghnaya et al. 2007; Małkowski et al. 2005), the considerably higher shoot N concentrations on HM_high_-sterilized soil when compared to HM_high_-nonsterilized soil was an unexpected result. We hypothesize that such differences between P_NS and P_S could be driven by a combined effect of HM toxicity and plant-microbe competition for mineral nutrients on the nonsterilized HM_high_ soil.

Higher concentration of Cd in shoots was found in both contaminated sterilized soils, whereas it was not observed in HM_low_ soil (G_S). This phenomenon may be associated with a low concentration of Cd in a bioavailable forms in uncontaminated soil. Distinct results were observed for Zn shoot concentrations, where significantly higher concentrations were obtained only in soil where Zn concentration was the highest (P_S). There are many reports which show the impact of AMF on reduction of HM translocation due to their immobilization on root surface by AMF hyphae (Abdelmoneim et al. 2014, Ban et al. 2015, Wu et al. 2016). Thus, it is supposed that observed in our current study, the higher concentration of Cd and Zn (only for P_S) in shoots of maize grown on sterilized and contaminated soils is a result of very low root colonization by AMF due to soil sterilization.

Direct measurements of photosynthetic rate clearly indicated an inhibition of this process due to an accumulation of HM in plant tissues, which has been widely reported (Farooq et al. 2013; Hattab et al. 2009; Mobin and Khan 2007). Slightly higher photosynthetic rates and higher F_M_ in plants grown on the P_S (HM_high_) soils when compared to the B_S (HM_mod_) could be related to higher N accumulation in shoots. Previous studies have suggested a correlation between chlorophyll content and N concentration in maize shoots (Schlemmer et al. 2013), which is with agreement to results obtained for chlorophyll content at the 45th day and the N concentration at the end of the experiment. Considerably higher concentrations of mineral nutrients, particularly N, could slightly mask the toxic effect of HM on the photosynthetic apparatus from sterilized HM_high_ soils (P_S). The differences between trends obtained for direct photosynthetic rate (A) and the efficiency of the light-dependent photosynthesis phase, obtained from chlorophyll a fluorescence analysis, may be associated with a higher impact of HM on enzymatic activity in the light independent phase when compared to its influence on the electron transport chain during light-dependent phase (Dias et al. 2013; Rodriguez et al. 2015).

Despite the fact that slight differences appeared at absorption energy flux per active reaction center (ABS/RC) and dissipated energy flux per reaction center (DIo/RC) between contaminated and uncontaminated variants, which influenced on the other parameters (φPo, PI_ABS_, PI_total_), no differences were found in further electron transport chain at parameters related to flux of excitation energy trapped per active RC (TRo/RC) and electron transport flux per RC (ETo/RC) as well as on JIP-test parameters related to the PSI functionality (data not shown). Interestingly, OJIP-test parameters indicated that a more pronounced effect was found when comparing sterilized with nonsterilized experimental variants. However, within this comparison, changes were only visible between uncontaminated (HM_low_) variants (G_NS vs. G_S) and highly contaminated (HM_high_) variants (P_NS vs. P_S). This observation may suggest that the competition between plant and microorganisms has a greater effect on plant growth and photosynthesis than the toxic effect of heavy metals.

This hypothesis explaining the slower growth of plants due to competition for nutrients between plants and autochthonous microorganisms seems to be reasonable taking into account the chlorophyll fluorescence analysis. This technique was previously used to assess the effect of nutrient deficiencies in the medium where plants were cultivated (Cetner et al. 2017; Kalaji et al. 2014; Kalaji et al. 2018; Tang et al. 2012). Kalaji et al. 2018 reported for rapeseed plants cultivated in soil with different mineral nutrients concentration that deficiencies of nutrients resulted in increase in ABS/RC even at low nutrients deficiencies; moreover, the authors found that there was increase in the K band at O-J phase. Those results are in agreement with phenomena observed between sterilized and nonsterilized soils in our study, i.e., nonsterilized soils may suffer a slight deficit due to competition. A decrease in the number of active reaction centers (RC) was considered as the mechanism of nutrient-deficient leaves against photo-oxidative damage and an excess of absorbed light energy, while the appearance of the K band denoted damage to the oxygen-evolving complex (Kalaji et al. 2014; Kalaji et al. 2018).

Transpiration rate, as well as stomatal conductance, decreased during the experiment, irrespectively of HM accumulation. The same trend was observed by Ci et al. (2010) and Anjum et al. (2016, 2017) for plants treated with different concentrations of Cd. The concentration of intracellular CO_2_ increased with the aging of plants and accumulation of HMs, which corresponds with results obtained by Tian et al. (2012). It has been shown that Cd concentration decreases the activity of enzymes involved in carboxylation reactions (e.g., RuBPCase) (Siedlecka et al. 1998), which could be related to an increased concentration of intracellular CO_2_. Values recorded for plant pigments and gas exchange parameters on the 18th and 32nd day could indicate the plants striving for an equilibrium state during acclimation in soil to varying environment conditions. This phenomenon is most visible in chlorophyll and anthocyanins content and photosynthesis rate. On the 18th day, sampling plant pigments did not differ significantly, while by the 32nd day, those parameters gave values characteristic for HM impact on plants, especially on sterilized experimental variants, while on nonsterilized variants this equilibrium was shifted in time. A similar phenomenon was observed for photosynthesis rate; however, on the 18th day, the highest values were recorded for plants cultivated on Bytom soils and the lowest for plants cultivated in Piekary Śląskie soils. On the 32nd day, the photosynthesis rate seems to stabilize at the levels which can suggest a toxic effect of HM on photosynthetic apparatus efficiency (Farooq et al. 2013). This phenomenon could be associated with a delay in plant reaction to the harsh environment due to reaching a critical concentration of ROS after some time from acclimation. It agrees with the suggestion by Poschenrieder et al. (2013) that metal induced ROS production can trigger antioxidant defenses that induce hormetic responses under certain conditions. However, once toxicity thresholds are passed, the ROS-induced scavenging system will be overcome and the negative effects on the antioxidant system and growth inhibition will occur.

The most common indicators of oxidative stress in plants are contents of H_2_O_2_ and malonedialdehyde (MDA) (Bidar et al. 2007; Rizwan et al. 2017; Wu et al. 2017; Zhang et al. 2007). However, there is also evidence for an increase of anthocyanin content under HM stress (Chen et al., 2015; Mobin and Khan 2007). Our results show clearly that cultivation of *Z. mays* on contaminated soil results in increased levels of H_2_O_2_, MDA and anthocyanin in leaves, corresponding to HM concentration in soil. Moreover, anthocyanin measurements suggest that differences between experimental variants appears earlier in sterilized treatments with anthocyanin content being lower in comparison to the nonsterilized treatments. Lower values of MDA and H_2_O_2_ concentrations in leaves were also obtained for sterilized soils at the end of the experiment. Zhang et al. (2007) reported that H_2_O_2_ accumulation in clover roots occurred in response to colonization by AMF. The similar tendencies obtained for MDA are likely driven by strong connectivity between H_2_O_2_ concentration and lipid peroxidation (Shahid et al. 2014).

Based on the results obtained from PCA, it could be assumed that autochthonous microorganisms are key players driving differences between plants cultivated in soil with different levels of contamination. In addition, differences between sterilized and nonsterilized variants were more apparent when higher microbial activity in the nonsterilized treatment was present, possibly demonstrating competition between plants and microorganisms.

## Conclusions

Our results showed that steam sterilization did not have a significant effect on soil physicochemical properties. The average percentage of colonization of root segments by arbuscular mycorrhiza fungi (AMF) decreased with the increase of heavy metals (HM) concentration in soil by more than 50% depending on the level of contaminants. Alongside a decrease in AMF colonization due to sterilization there was significant increase in Cd shoot concentration by about 119 and 228% for B_S and P_S variants, respectively, when compared to nonsterilized counterparts. However, Zn plant shoot concentration was significantly higher by 68% only for P_S variant, when compare to nonsterilized counterpart. Dark septate endophytes were only present in roots of plants cultivated in HM_high_ soils. Lower biomass yields on nonsterilized soil could be the result of competition between plants and microorganisms for mineral nutrients. Anthocyanins content could be good indicator of HM-induced oxidative stress due to its correlation with MDA and H_2_O_2_ leaf content. Autochthonous microflora seems to be a key player in determining differences between *Zea mays* physiological status cultivated on the sterilized and nonsterilized contaminated soils, having a negative impact on growth and photosynthetic performance, likely due to competition with plants for nutrients. This observation tends to overthrow the hypothesis that autochthonous microorganisms improve plant growth and photosynthetic performance. However, a lack of these microorganisms in the same soils considerably increased plants HM concentration, particularly Cd and Zn.

## Electronic supplementary material

ESM 1 (DOCX 2.22 mb)
