# Supplementary material for: How autochthonous microorganisms influence physiological status of Zea mays L. cultivated on heavy metal contaminated soils?
Source: Environ Sci Pollut Res Int. 2018 Dec 18;26(5):4746–63. doi: 10.1007/s11356-018-3923-9 (PMC6394448; doi:10.1007/s11356-018-3923-9)
Supplement: Supplementary file 1 — (DOCX 2.22 mb) [file 11356_2018_3923_MOESM1_ESM.docx]

**Supporting Information - How autochthonous microorganisms influence physiological status of Zea mays L. cultivated on heavy metal contaminated soils?**


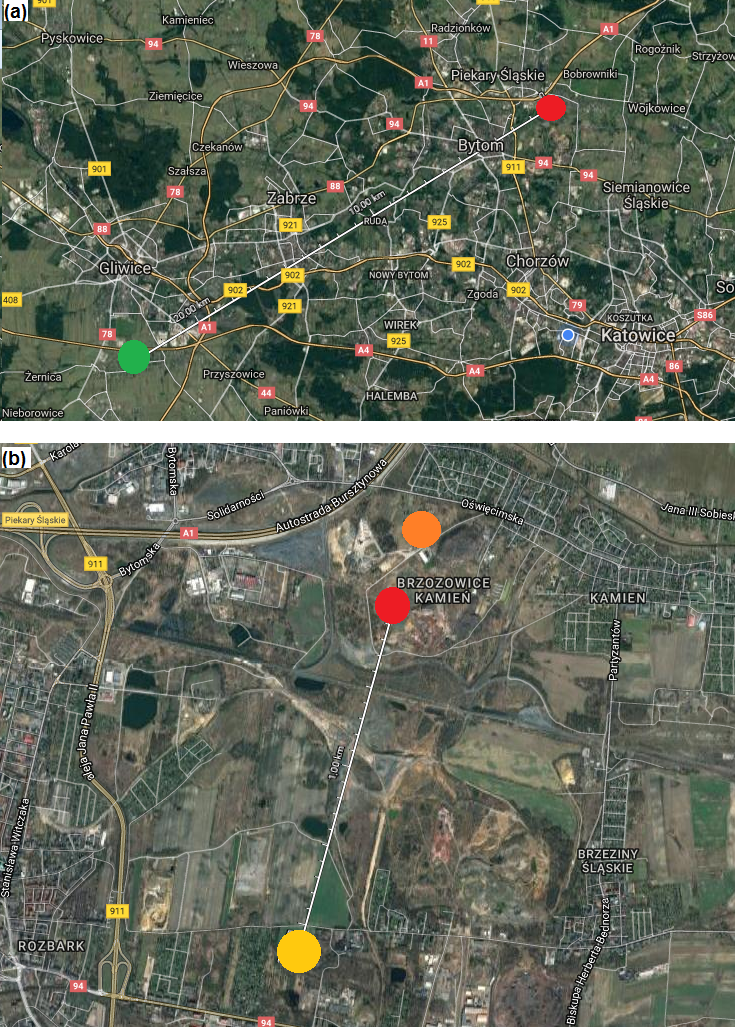


**Fig. S1** Soil collection points. Red circle (a, b) – localization of close down Pb/Zn smelter, Green circle (a) – localization of uncontaminated site (G, HM_low_), Yellow circle (b) – localization of moderately contaminated site (B, HM_mod_), Orange circle (b) – localization of highly contaminated site (P, HM_high_). Source: https://www.google.pl/maps - modified


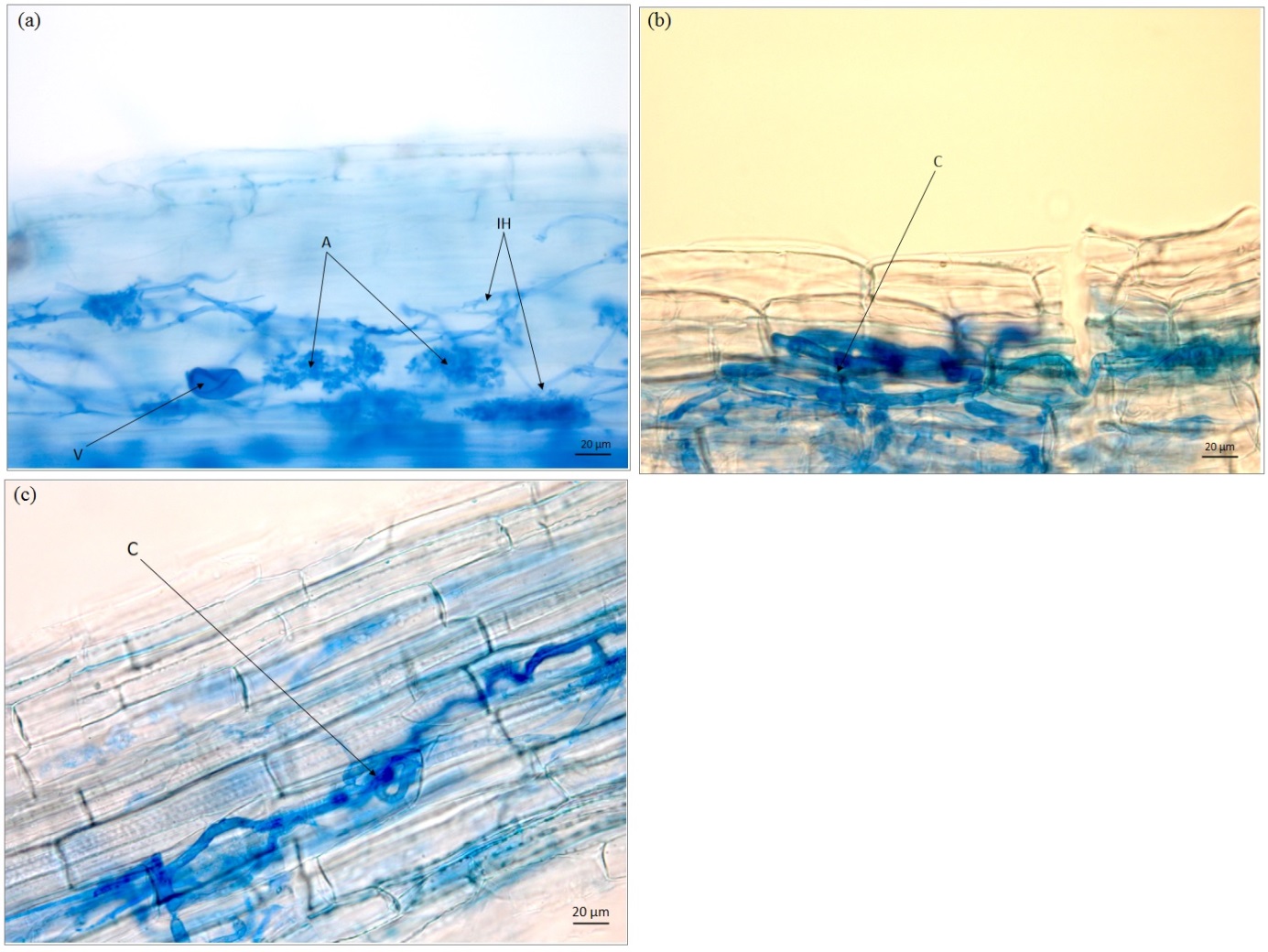


**Fig. S2** Arbuscular mycorrhizal structures in *Zea mays* roots collected from the different experimental variants. (a) G_NS – roots obtained from uncontaminated unsterilized soil, (b) B_NS – roots obtained from moderately contaminated unsterilized soil, (c) P_NS – roots obtained from highly contaminated unsterilized soil. A – arbuscules, V – vesicles, C – coils, IH – internal hyphae


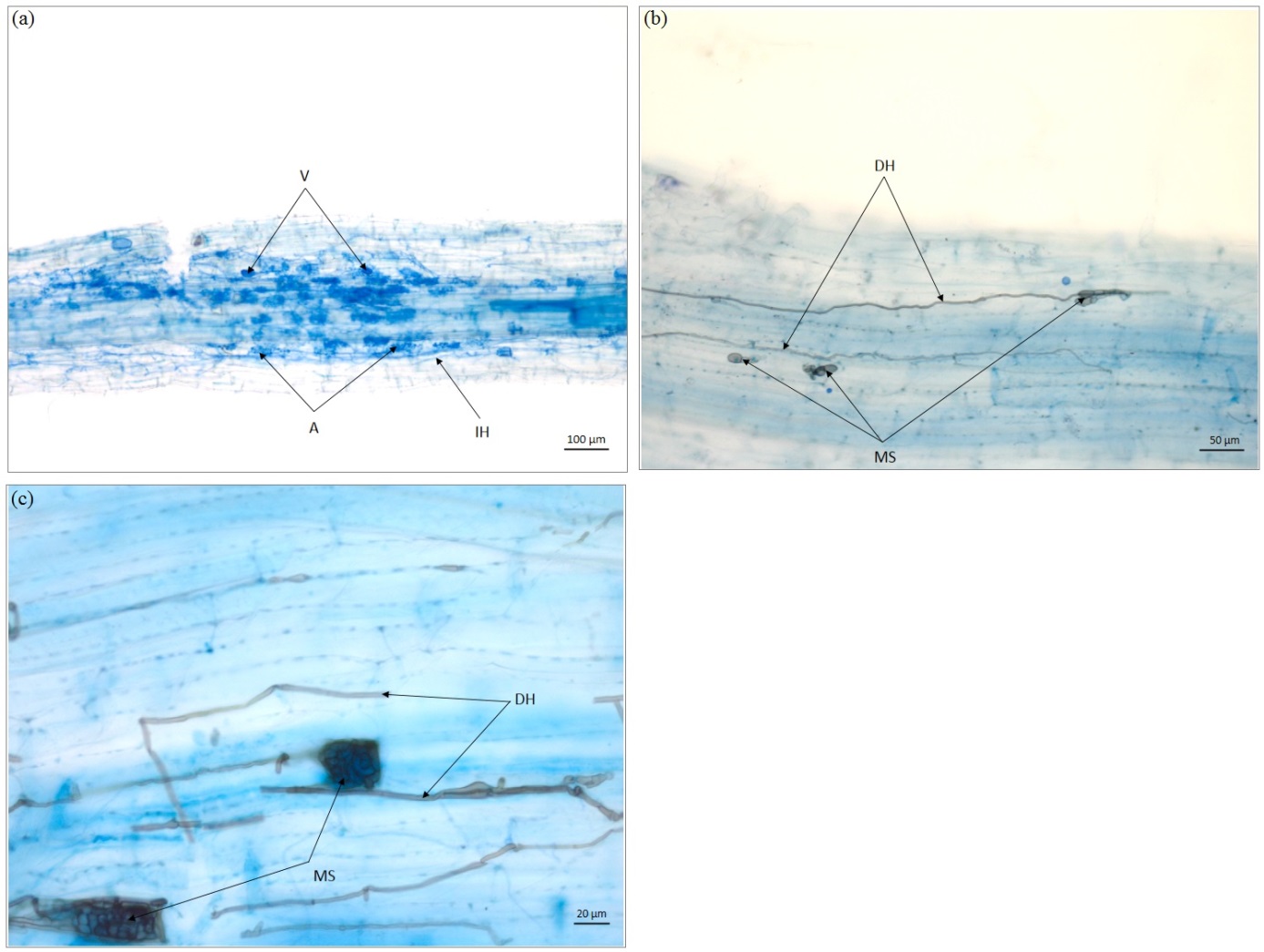


**Fig. S3** Dark septate endophyte (DSE) structures and arbuscular mycorrhizal structures in *Zea mays* roots collected from the different experimental variants. (a) G_NS – roots obtained from uncontaminated unsterilized soil (b) B_NS – roots obtained from moderately contaminated unsterilized soil, (c) P_NS – roots obtained from highly contaminated unsterilized soil. A – arbuscules, V – vesicles, C – coils, IH – internal hyphae, MS – microsclerotiam, DH – DSE hyphae

**Table S1** Results of hydrometric soil texture analysis of soil samples

| Fraction | Fraction size (mm) | Experimental variants | | | | | |
| --- | --- | --- | --- | --- | --- | --- | --- |
|  |  | G_NS | G_S | B_NS | B_S | P_NS | P_S |
|  |  | Percentage of fraction (%) | | | | | |
| Silt | < 0.002 | 40 ± 5.1 | 39 ± 3.4 | 25 ± 3.4 | 25 ± 1.6 | 7 ± 0.2 | 7 ± 0.3 |
| Clay | 0.002 – 0.05 | 45 ± 8.1 | 48 ± 7.8 | 60 ± 3.9 | 59 ± 4.8 | 75 ± 0.9 | 74 ± 1.5 |
| Sand | 0.05 – 2.0 | 15 ± 5.5 | 13 ± 4.4 | 15 ± 0.5 | 16 ± 3.1 | 18 ± 1.0 | 19 ± 1.6 |

Values are means ± SE (n = 3)
